# Postmastectomy radiation therapy in breast cancer patients with micrometastatic disease in sentinel node dissection: A cohort study and *meta*-analysis

**DOI:** 10.1016/j.ctro.2024.100770

**Published:** 2024-03-25

**Authors:** Fatema Jafer, Stilda Malki, Mariam Akram, Tamana Gulwarisdotter, Andreas Karakatsanis, Antonis Valachis

**Affiliations:** a Faculty of Medicine and Health, Örebro University, Örebro, Sweden; b Department of Oncology, Faculty of Medicine & Health, Örebro University, 70182, Örebro, SE, Sweden; c Department of Surgical Sciences, Uppsala University, Uppsala, Sweden

**Keywords:** Breast cancer, Mastectomy, Micrometastasis, Sentinel lymph node dissection, Radiation therapy, Cohort, Meta-analysis

## Abstract

**Aim:**

The potential role of postmastectomy radiation therapy (PMRT) on prognosis in patients with T1-2 breast cancer and micrometastatic disease in sentinel lymph node dissection (SLND) has not yet been established. The aim of this study was to investigate the impact of PMRT on prognosis in patients with T1-2 breast cancer and micrometastatic in SLND.

**Method:**

A register- and population-based cohort was utilized by identifying eligible patients on the research database BcBase 3.0. Multivariate Cox regression models were applied for survival outcomes. In addition, a systematic literature review and *meta*-analysis including all relevant studies on this topic was performed.

**Results:**

In total, 956 patients fulfilling the inclusion criteria were found through the BcBaSe 3.0 with 237 (25.0 %) receiving PMRT and 719 (75.0 %) not receiving PMRT. No statistically significant differences between the two patient groups in terms of neither breast cancer-specific (adjusted Hazard Ratio (HR): 0.49; 95 % Confidence Interval (CI): 0.14 – 1.73) nor overall survival (adjusted HR: 0.63; 95 % CI: 0.29 – 1.35) was found. In the pooled analyses after literature review, PMRT did not result in better breast cancer-specific (5 studies; pooled HR: 1.06; 95 % CI: 0.88–1.27; I^2^ = 1 %; low certainty of evidence) or overall survival (6 studies; pooled HR: 1.01; 95 % CI: 0.91–1.13; I^2^ = 10 %; low certainty of evidence).

**Conclusion:**

PMRT does not seem to impact survival in patients with T1 or T2 breast cancer with micrometastatic disease in SLND. Considering the low level of evidence and the relatively short follow-up of included studies, caution in interpreting the results into clinical practice is suggested.

## Highlights

• The role of postmastectomy radiotherapy in micrometastatic axillary disease is questionable.
• Postmastectomy radiotherapy did not result in better survival in the register-based cohort.
• No survival benefit with postmastectomy radiotherapy was found in *meta*-analysis either.
• This treatment strategy does not seem to impact survival in patients with micrometastatic axillary disease.

## Introduction

Although breast conserving surgery (BCS) with adjuvant radiation therapy (RT) has been proven to be equivalent in terms of survival compared to mastectomy [1] with lower risk for complications [2], a considerable number of patients with early breast cancer still undergo mastectomy [3]. The role of postmastectomy RT (PMRT) is less studied compared to RT after BCS but international guidelines suggest using RT in large tumors (>5 cm) or in axillary lymph node metastases irrespective of the surgical procedures, namely sentinel lymph node dissection (SLND) or axillary dissection [4], [5], [6].

There is, however, a clinical situation where the potential impact of PMRT remains controversial. In fact, in tumors less than 5 cm where mastectomy has been performed and only micrometastatic disease has been found in the SLND, the potential benefit of PMRT has not been proven. The current evidence comprises observational studies with a relatively limited number of patients and somewhat conflicting results [7], [8], [9], [10], [11], [12], [13].

The aim of the present study was to investigate the potential impact of PMRT on prognosis in patients with T1-2 tumors and micrometastatic disease in sentinel lymph nodes by utilizing a register- and population-based cohort and then applying a *meta*-analysis including all relevant published evidence on the topic.

## Methods

### Settings, sources, definitions, and outcomes of cohort study

The registry-based research database, Breast Cancer Data Base Sweden (BCBaSe) 3.0, was used to collect data for the retrospective cohort study. BCBaSe 3.0 is based on a linkage between the National Quality Register for breast cancer, collecting data regarding all newly diagnosed breast cancer patients in Sweden, and other Swedish national databases of interest as the Swedish Cause of Death Register, the Swedish National Patient Register, and the longitudinal integrated database for health insurance and labour market studies (LISA) [14].

We included all patients diagnosed with T1-2 breast cancer between 2008 and 2020 who had undergone mastectomy and SLND only, with micrometastatic disease in the specimen. We excluded patients with *de novo* metastatic disease, those treated with neoadjuvant therapy, and patients who underwent axillary dissection after SLND.

The primary outcome for the study was breast cancer-specific survival (BCSS), defined as the time from breast cancer diagnosis until death due to breast cancer. Secondary outcome was overall survival (OS), defined as the time from breast cancer diagnosis until death from any cause. Since the validity of recurrence data in National Quality Registry for breast cancer seems to be lower compared to survival outcomes, we avoided analyzing recurrence-related outcomes, such as disease-free survival.

### Search strategy, study selection and data collection process for meta-analysis

The present systematic review and *meta*-analysis is reported in accordance with the MOOSE (Meta-analysis Of Observational Studies in Epidemiology) guidelines [15].

The electronic databases PubMed, Web of Science, and Cochrane were used as sources to identify relevant articles. The search algorithm utilized the following keywords in different combinations: micrometasta*, occult, radiotherapy, radiation therapy, breast cancer, breast tumor, breast malign*. No year restriction was set but only studies published in English were retrieved. The last search was performed on 30 April 2023.

The PICO approach was applied to define inclusion and exclusion criteria for potentially eligible studies.

(P)opulation: T1 or T2 patients who underwent mastectomy and had micrometastasis in SLND.

(I)ntervention: PMRT.

(C)omparison: No RT.

(O)utcome: Locoregional-recurrence free survival, disease-free survival, breast cancer-specific survival, overall survival, or lymphedema.

We excluded studies that did not present separate data on patients treated with mastectomy and patients with micrometastatic disease in ALND. We also excluded studies without comparative results between patients receiving PMRT and those without RT. Finally, we excluded studies that presented comparative results between PMRT and no RT without applying any suitable statistical methodology (multivariate analyses, propensity score approaches) to mitigate confounding by indication bias.

The study selection process was performed independently by two researchers and a third researcher resolved any issues.

The following data were collected from eligible studies: first author’s surname, year of publication, journal, country of origin, type of study (retrospective vs. prospective), number of study sites, patient inclusion period, total number of included patients, median follow-up for survival outcomes, adjusted hazard ratios (HR) and their corresponding 95 % confidence intervals (CI) for each survival outcome presented, variables included in the multivariate models for each analysis, and methodological aspects for assessment of study quality according to the Newcastle-Ottawa scale (NOS; [16]).

Two researchers collected data independently from eligible studies using a pre-defined form. Any discrepancies were resolved by a third researcher.

The quality of the included articles was assessed using the NOS for cohort studies investigating three general methodological aspects, namely selection, comparability, and outcome. Based on these aspects, up to nine stars can be assigned to each study with more stars indicating higher study quality.

To assess the quality of evidence for pooled results from *meta*-analysis, the GRADE approach was applied [17].

### Statistical analysis and data synthesis

For the cohort study, categorical variables are summarized as numbers and percentages whereas continuous variables as median and range. For comparisons of patient- and tumor-related characteristics between patients treated with PMRT and those without RT, chi-square test was used for categorical variables and Mann-Whitney test for continuous variables. To investigate the impact of postmastectomy RT on time-to-event outcomes (BCSS, OS), multivariate Cox regression models were used using the following variables as covariates: age, household income, tumor grade, pathological T status (T1 vs. T2), estrogen-receptor status (ER-status), HER2 status, use of adjuvant chemotherapy, and use of adjuvant endocrine therapy.

For *meta*-analysis, all study-level HR and corresponding 95 % CI from multivariate analyses for each survival outcome of interest were converted to logHR and standard error. The inverse-variance method was applied for calculation of pooled HR and 95 % CI. The statistical heterogeneity was illustrated through I^2^ with a 50 % cut-off to be considered as high heterogeneity. In the presence of heterogeneity, random-effects model was used to pool the data whereas in the absence of heterogeneity, fixed-effects model was applied.

The analyses for cohort study were performed using SPSS (version 21) whereas the Review Manager (version 5.3) was used for *meta*-analysis.

## Results

### Characteristics of study cohort

Through the BCBaSe 3.0 database, 956 patients with breast cancer fulfilling all the inclusion criteria were identified of whom 237 (25 %) received PMRT and 719 (75 %) did not receive PMRT. The target volume of PMRT included chest wall without regional irradiation but no information on PMRT schedule was retrieved.

Patients treated with PMRT tended to be younger, with higher income, and biologically more aggressive breast cancer thus leading to more adjuvant systemic therapy as well. Some geographical differences were also observed. The baseline characteristics of the study cohort with comparisons between PMRT and no RT are presented in Table 1.

**Table 1:** Baseline characteristics of study cohort.

	PMRT n (%)	No-PMRT n (%)	P-value
Total N of patients	237	719	
Age, median (range)	54 (34–81)	69 (24–95)	< 0.001
Sex Female Male	230 (97) 7 (3)	700 (97.4) 19 (2.6)	0.798
Healthcare region Northern Stockholm Gotland Uppsala-Örebro South Southeast Western	20 (8.5) 64 (27.1) 65 (27.5) 23 (9.7) 37 (15.7) 27 (11.4)	69 (9.6) 138 (19.3) 159 (22.2) 159 (22.2) 69 (9.6) 122 (17)	< 0.001
Household income (quartiles) Q4 (lowest) Q3 Q2 Q1 (highest)	48 (20.3) 45 (19.1) 53 (22.5) 90 (38.1)	238 (33.2) 182 (25.4) 153 (21.4) 143 (20)	< 0.001
Tumor size T1 T2	91 (38.4) 146 (61.6)	337 (46.9) 382 (53.1)	0.023
Estrogen receptor status Positive Negative	214 (90.3) 23 (9.7)	649 (90.5) 68 (9.5)	0.920
HER2-status Positive Negative	33 (13.9) 201 (84.9)	101 (14.0) 594 (82.6)	0.245
Tumor grade I II III	16 (6.8) 143 (60.9) 76 (32.3)	106 (15.0) 395 (55.9) 205 (29.0)	0.005
Adjuvant chemotherapy	155 (65.4)	212 (29.5)	< 0.001
Adjuvant endocrine therapy	210 (88.6)	528 (73.4)	< 0.001

### Impact of PMRT on breast cancer-specific and overall survival in study cohort

The median follow-up for the cohort was 62 months. In terms of both breast cancer-specific and overall survival, PMRT did not result in a statistically significant improved outcome when utilizing multivariate Cox regression model for adjustments (adjusted HR for breast cancer-specific survival: 0.49; 95 % CI: 0.14–1.73; adjusted HR for overall survival: 0.63; 95 % CI: 0.29 – 1.35).

### Study selection, characteristics, and quality assessment of eligible studies for meta-analysis

**Fig. 1:**
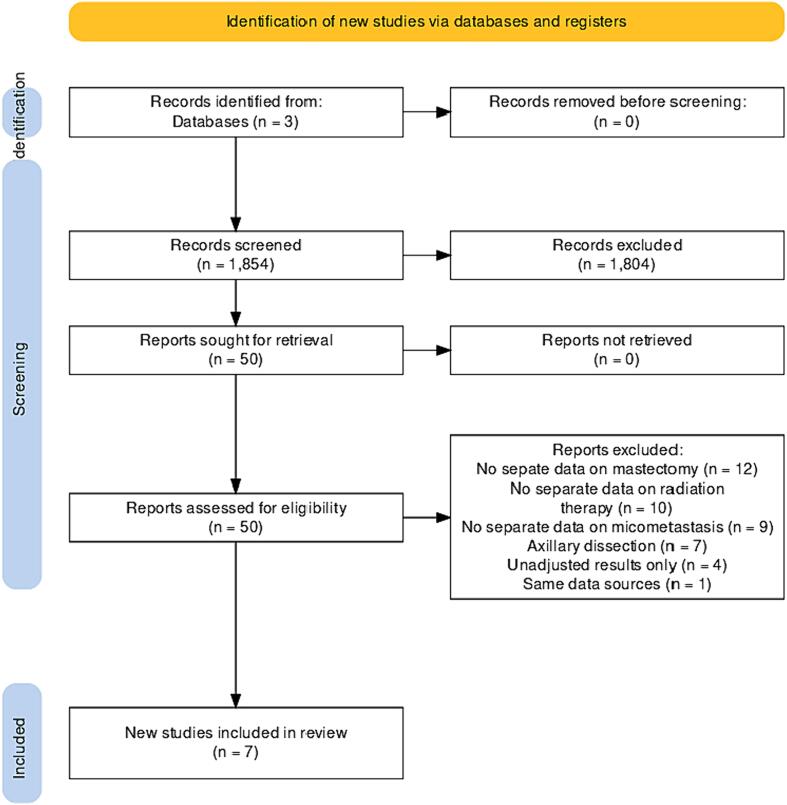
Flowchart of study selection process for *meta*-analysis.

Through the literature review of initially 1854 articles, 7 studies met all the inclusion criteria. A flowchart of the study selection process is shown in Fig. 1.

The characteristics of eligible studies are presented in Table 2. Five studies were register-based and two institutional-based retrospective cohort studies. The median follow-up ranged between 50.4 and 60.8 months whereas two studies did not report median follow-up time. Three studies applied a propensity score approach for mitigating confounding by indication bias whereas all studies used Cox regression models for their analyses.

**Table 2:** Characteristics of eligible studies for *meta*-analysis.

**Author, Year (ref)**	**Country**	**Study type**	**N pts**	**N pts with postmastectomy RT**	**Postmastectomy RT volume and schedule**	**Median follow-up (months)**	**Outcomes**	**Propensity score analysis**
Luo, 2022 [7]	USA	Register-based retrospective cohort	2864	588	NR	53	BCSS, OS	Yes
Merfeld, 2022 [8]	USA	Institutional-based retrospective cohort	156	19	72 % chest wall only; 28 % regional nodal irradiation	60.8	LRFS	No
Patel, 2020 [9]	USA	Register-based retrospective cohort	5878	1202	NR	NR	BCSS, OS	Yes
Shi, 2020 [10]	USA	Register-based retrospective cohort	4729	1062	NR	NR	BCSS	No
Picado, 2018 [11]	USA	Register-based retrospective cohort	4858	NR	NR	53	OS	No
Wu, 2018 [12]	USA	Register-based retrospective cohort	14,019	2651	NR	53.1	OS	Yes
Forissier, 2017 [13]	France	Institutional-based retrospective cohort	134	98	73 % chest wall only; 27 % regional nodal irradiation	50.4	LRFS, DFS, BCSS, OS	No

*Abbreviations*: RT, radiotherapy; OS, overall survival, LRFS, locoregional recurrence-free survival; NR, not reported; BCSS, breast cancer-specific survival; DFS, disease-free survival.

In terms of patient-, tumor-, and treatment-related characteristics among eligible studies, between-study heterogeneity was observed mainly in adjuvant treatment patterns (Supplementary Table 1).

The quality assessment of eligible studies is presented in Table 3. All studies were rated with at least 7 stars, but none reached 9 stars.

**Table 3:** Quality assessment of eligible studies using the Newcastle-Ottawa scale.

Author (ref)	Selection				Comparability		Outcome			Total score
	Representativeness of exposed cohort	Selection of non-exposed	Ascertainment of exposure	Outcome of interest not present at study start	Regarding age (as the most important factor)	Regarding other prognostic factors	Assessment	Long enough follow-up (at least 8 years)	Adequacy of follow-up (<10 % lost-to-follow-up)	
Luo [7]	*	*	*	*	*	*	*	–	–	7
Merfeld [8]	*	*	*	*	*	*	*	–	–	7
Patel [9]	*	*	*	*	*	*	*	–	–	7
Shi [10]	*	*	*	*	*	*	*	–	–	7
Picado [11]	*	*	*	*	*	*	*	–	–	7
Wu [12]	*	*	*	*	*	*	*	–	*	8
Forissier [13]	*	*	*	*	*	*	*	–	–	7

### Pooled analyses on the impact of PMRT on survival outcomes

Two studies [8], [13] presented results regarding the impact of PMRT on locoregional recurrence. Merfeld et al. (8) found a statistically significant improved locoregional recurrence-free survival in patients treated with PMRT (HR: 0.22; 95 % CI: 0.06 – 0.81), whereas no statistically significant difference was found in Forissier et al. [13] (HR: 0.45; 95 % CI: 0.18 – 1.09). Both studies included a limited number of patients. One study presented data on disease-free survival and found no statistically significant difference between patients treated with RT and those without RT (HR: 0.61; 95 % CI: 0.36 – 1.03). No pooled analyses were performed for these outcomes.

**Fig. 2:**
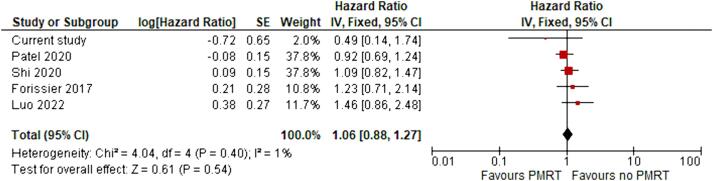
Pooled hazard ratio and 95% confidence interval for breast cancer-specific survival between patients with pT1-2pN1mi breast cancer who underwent mastectomy and sentinel node dissection treated with postmastectomy RT and those without RT.

Five studies (14 561 patients), including the current cohort, presented data on breast cancer-specific survival. The pooled HR was 1.06 (95 % CI: 0.88–1.27; I^2^ = 1 %) indicating no difference between the two treatment groups in terms of breast cancer-specific survival (Fig. 2).

**Fig. 3:**
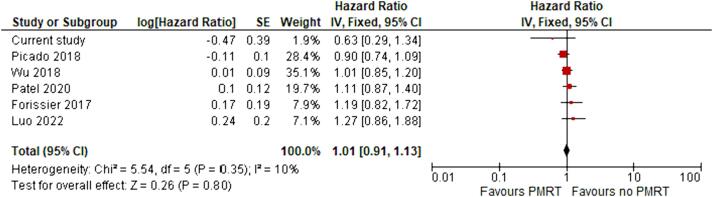
Pooled hazard ratio and 95% confidence interval for overall survival between patients with pT1-2pN1mi breast cancer who underwent mastectomy and sentinel node dissection treated with postmastectomy RT and those without RT.

Six studies (28 709 patients), including the current study, presented data on overall survival. No statistically significant difference in terms of overall survival between patients treated and those not treated with PMRT was observed (pooled HR: 1.01; 95 % CI: 0.91 – 1.13; I^2^ = 10 %; Fig. 3).

### Impact of PMRT on lymphedema

We found no study reporting the impact of PMRT on lymphedema compared to no RT.

### Grading the level of evidence

Using the GRADE approach, we found that the certainty of evidence for both breast cancer-specific and overall survival was low mostly due to the risk for confounding by indication bias in all eligible studies and the retrospective nature of all eligible studies that precludes any causal association in the results (Table 4). Limited evidence was observed in two relevant survival outcomes (locoregional recurrence-free survival, disease-free survival) whereas no evidence on the impact of PMRT on lymphedema in this clinical situation was found.

**Table 4:** GRADE approach for assessing the certainty of evidence from pooled analyses.

Outcomes	Relative effect(95 % CI)	№ of participants(studies)	Certainty of the evidence(GRADE)	Comments
Locoregional recurrence	not estimable	290 (2 non-randomised studies)	N/A	Only 2 studies; unable to perform pooled analysis
Disease-free survival	not estimable	134 (1 non-randomised study)	N/A	Only 1 study; unable to perform pooled analysis
Breast cancer specific survival	HR 1.06 (0.88 to 1.27)	14561(4 non-randomised studies)	⊕⊕○○ Low	High risk for confounding by indication bias in all eligible studies despite the use of suitable statistical methods
Overall survival	HR 1.01 (0.91 to 1.13)	28709(5 non-randomised studies)	⊕⊕○○ Low	High risk for confounding by indication bias in all eligible studies despite the use of suitable statistical methods
Lymphedema	not estimable	0 (0 studies)	N/A	Completely lack of evidence

## Discussion

In the present cohort study and *meta*-analysis of retrospective cohorts, we found that PMRT in patients with T1 or T2 breast cancer and micrometastatic disease in SLND treated with mastectomy without axillary dissection does not seem to impact prognosis. The quality of evidence is, however, low and, for some relevant outcomes as locoregional recurrence and lymphedema, is lacking, thus underlining the need for high quality evidence using study designs that can support assessment of causal associations.

The current evidence is limited by the risk of biases accompanying the methodological nature of published studies on this topic. First, the risk of confounding by indication should be acknowledged as an important limitation in comparative retrospective studies [18]. In fact, we found substantial differences in patient- and tumor-related characteristics between patients treated with PMRT compared to those without RT in our cohort, as younger patients with unfavorable anatomical and biological tumor characteristics seemed to receive RT more often. This trend was evident in other cohorts as well [7], [8], [9], [10], [11], [12] suggesting a high risk for confounding by indication bias. To mitigate this risk, we performed multivariate analyses in our study cohort, and we considered studies as eligible to pooled analyses only if they applied suitable statistical methods to mitigate the bias.

Another issue that should be considered when interpreting the results is that the median follow-up in all including studies was approximately five years. This time frame should be considered relatively short for studies where most patients had an ER-positive/HER2-negative disease where late recurrence is the rule of thumb [19]. As a result, study cohorts with longer follow-up to enable the analysis of late recurrences are needed. An additional issue with the current results is the fact that the same data sources, with some overlapping in time periods for patient inclusion, were used in some of the studies [7], [10], [11], [12] and, therefore, there is a risk for some duplicates in terms of included patients in pooled analyses.

Regarding the outcomes of interest that were able to be presented, our analyses were restricted to breast cancer-specific and overall survival due to the lack of adequate data for locoregional recurrence-free and disease-free survival that could also be of interest for this patient population. In addition, the potential impact of PMRT on the occurrence of lymphedema in this clinical scenario has not been evaluated. As radiation therapy to axillary lymph nodes seems to contribute to an increased risk for lymphedema [20], this outcome should also be included as of interest for a complete and more balanced risk–benefit assessment.

Another issue that deserves attention is the lack of information on the PMRT volume and schedule among different eligible studies as well as the observed between-study heterogeneity in terms of adjuvant treatment patterns. The latter shortcoming could partially be explored and mitigated by suitable statistical analyses in eligible studies and in pooled analyses. However, the lack of information about PMRT volume poses challenges in the interpretation of results as target volume can influence the outcome. In our study cohort as well as in eligible studies that include this information, target volume was mainly defined as chest wall without intentional irradiation of axillary lymph nodes. This information should be considered when interpreting the results of the *meta*-analysis.

The study results are somewhat contradictory to the prospective non-randomized study SENOMIC where a higher risk for recurrence was implied in patients treated with mastectomy without PMRT [21]. However, the non-randomized design of SENOMIC poses similar challenges regarding the risk for confounding by indication as in the retrospective studies and the lack of statistical efforts to mitigate this risk negatively influences the reliability of the results. Additionally, a sub-analysis of SINODAR-ONE trial where patients with mastectomy due to cT1-2 disease and no more than two sentinel lymph nodes with macrometastases were randomized to ALND or not showed similar outcomes between the two arms even if only 17 % of patients received PMRT [22].

The lack of randomized evidence on the role of PMRT in patients with low-volume axillary disease, defined as the presence of 1 to 3 lymph nodes with micrometastatic disease, has been highlighted in a recent Cochrane-based systematic review where only one randomized trial utilizing modern-day RT was identified [23]. The latter showed a survival benefit with the addition of PMRT in patients with macrometastases in 1 to 3 axillary lymph nodes [24]. However, the study included only patients with macrometastatic disease treated with axillary dissection, thus not reflecting the patient population of the current study cohort and *meta*-analysis. Recently, an individual patient data *meta*-analysis from EBCTCG (Early Breast Cancer Trialists’ Collaborative Group) showed a survival benefit with the addition of radiotherapy to regional lymph nodes in all patients with breast cancer and lymph node metastases, irrespectively the surgical procedure to the breast (mastectomy or breast conserving surgery) or the number of positive lymph nodes [25]. However, no information on micrometastatic disease in sentinel lymph nodes was captured and analyzed in the EBCTCG *meta*-analysis and, as a result, the findings cannot be directly generalized to the patient population of the current study cohort.

Despite these caveats, the current study presents the current evidence using the GRADE approach, thus facilitating the clinicians to interpret the results based on the quality and certainty of evidence. Although the consistency of results across eligible studies can be argued to be a source of robustness of study results, the low level of evidence reflecting the retrospective nature of eligible studies suggest caution in interpreting the results into clinical practice.

In conclusion, PMRT does not seem to impact the survival in patients with T1 or T2 breast cancer and micrometastatic disease in SLND. Considering the low level of evidence associated with the retrospective nature of eligible studies leading to a high risk of bias, caution in interpreting the results into clinical practice is suggested until further evidence from prospectively collected and analyzed data using a broad spectrum of outcomes of interest, covering both survival and toxicity, for this clinical situation are available.

## CRediT authorship contribution statement

**Fatema Jafer:** Data curation, Methodology, Writing – original draft. **Stilda Malki:** Data curation, Methodology, Writing – original draft. **Mariam Akram:** Data curation, Methodology, Writing – original draft. **Tamana Gulwarisdotter:** Data curation, Methodology, Writing – original draft. **Andreas Karakatsanis:** Conceptualization, Methodology, Supervision, Validation, Writing – review & editing. **Antonis Valachis:** Conceptualization, Formal analysis, Methodology, Supervision, Validation, Visualization, Writing – review & editing.

## Declaration of competing interest

The authors declare that they have no known competing financial interests or personal relationships that could have appeared to influence the work reported in this paper.
