# Supplementary material for: Postmastectomy radiation therapy in breast cancer patients with micrometastatic disease in sentinel node dissection: A cohort study and meta-analysis
Source: Clin Transl Radiat Oncol. 2024 Mar 25;46:100770. doi: 10.1016/j.ctro.2024.100770 (PMC10998037; doi:10.1016/j.ctro.2024.100770)
Supplement: Supplementary data 1 [file mmc1.docx]

Supplementary Table 1. Patient-, tumor-, and treatment-related characteristics among eligible studies in meta-analysis

| **Author, Year** | **Median age** | | **Grade III, %** | | **Positive surgical margins, %** | | **LVI, %** | | **ER-status, %** | | **HER2-status, %** | | **Chemotherapy used** | | **Endocrine therapy used** | |
| --- | --- | --- | --- | --- | --- | --- | --- | --- | --- | --- | --- | --- | --- | --- | --- | --- |
|  | PMRT | No PMRT | PMRT | No PMRT | PMRT | No PMRT | PMRT | No PMRT | PMRT | No PMRT | PMRT | No PMRT | PMRT | No PMRT | PMRT | No PMRT |
| Luo, 2022* | 54 | 58 | 33.5 | 27.5 | NR | NR | NR | NR | 86.6 | 88.9 | NR | NR | 69.2 | 45.1 | NR | NR |
| Merfeld, 2022 | 55 | 50.5 | 22.0 | 32.6 | 0.9 | 6.4 | 22.0 | 19.1 | 85.3 | 93.6 | 15.6 | 14.9 | 47.7 | 48.9 | 85.3 | 76.6 |
| Patel, 2020* | 50 | 55 | 47 | 39 | NR | NR | NR | NR | 77 | 80 | 12 | 9 | 84 | 53 | NR | NR |
| Shi, 2020 | NR | NR | 40.0 | 29.8 | NR | NR | NR | NR | 84.4 | 89.0 | 17.4 | 13.9 | 77.4 | 46.1 | NR | NR |
| Picado, 2018** | NR | NR | NR | NR | NR | NR | NR | NR | NR | NR | NR | NR | NR | NR | NR | NR |
| Wu, 2018* | NR | NR | 35.4 | 28.2 | 8.9 | 2.6 | 27.9 | 18.2 | 84.0 | 86.5 | NR | NR | 77.8 | 53.0 | 79.5 | 74.1 |
| Forissier, 2017 | NR | NR | 26.0 | 17.0 | NR | NR | 29.1 | 14.4 | 85.4 | 85.1 | 16.2 | 13.4 | 54.9 | 28.3 | 75.2 | 67.1 |
| Current study | 54 | 69 | 32.3 | 29.0 | 0 | 0 | NR | NR | 90.3 | 90.5 | 13.9 | 14.0 | 65.4 | 29.5 | 88.6 | 73.4 |

Abbreviations: PMRT, postmastectomy radiotherapy; LVI, lymphovascular invasion; ER, estrogen-receptor; NR, not reported.

*Results are presented before propensity score matching

**No separate data on pNmi cohort are reported
